# Retraction: Effect of sensory art therapies on root canal treatment anxiety and high dental anxiety in adults: A systematic review with meta-analysis

**DOI:** 10.1371/journal.pone.0357077

**Published:** 2026-08-27

**Authors:** The PLOS One Editors

After publication of this article [1], concerns were raised about the methodology. Specifically:

- The authors used an umbrella term (sensory art therapies) for several highly variable interventions such as virtual reality, aromatherapy, music therapy, chromotherapy (color therapy), but also yoga and aerobic exercise. This heterogeneity makes it unclear which intervention type, if any, drives the observed effects, and limits the validity of the pooled analyses and the interpretability of the results.
- One study [2] included in the systematic review and meta-analysis [1] does not appear to meet the predefined participants (P) criterion of the PICOS framework specified in the Methods section. Specifically, the systematic review defined eligible participants as adult patients with symptoms related to dental root canal treatment, high dental anxiety, or dental phobia, whereas [2] also included patients undergoing routine dental care who were not necessarily receiving root canal treatment and did not necessarily have high dental anxiety or dental phobia, but rather a range of dental anxiety levels from none or low to high.
- Two studies [3,4] included in the systematic review [1] do not appear to meet the predefined intervention (I) criterion of the PICOS framework specified in the Methods section. Specifically, the systematic review defined eligible interventions as art therapy, including sensory based art therapies targeting the five senses, whereas [3] and [4] evaluated aerobic exercise and yoga, respectively, instead. These two studies were not included in the meta-analysis.
- The authors conducted eight outcome-specific meta-analyses, each based on a small number of studies (n = 2, 4, 2, 4, 3, 6, 6, 3; Figs 3 and 4), raising concerns about the robustness of the pooled estimates. This concern is further compounded by the inclusion of multiple subgroups from the same studies in six outcomes, which may disproportionately influence the results.
- There is a discrepancy in the GRADE rating for heart rate (HR) between Fig 5 (moderate) and Table 5 (high). Additionally, editorial follow-up with the first author revealed that certainty of evidence was upgraded for visual analogue scale (VAS), HR, and salivary cortisol (SC) on the basis of effect size magnitude. According to the Cochrane GRADE Book, however, upgrading on this basis is not permitted when the included studies are RCTs. Correcting for this, the certainty of evidence range changes from the reported “very low to high” to “very low to low.”
- The study in [5] was included as four entries (a-d) in Fig 4 for both systolic and diastolic blood pressure, each compared against the same 30-person control group. The first author clarified that these four entries correspond to two intervention groups (Chinese and Western music), each measured at two appointments within a single root canal treatment course. Treating repeated measurements on the same individuals, compared repeatedly against the same controls, as independent data points violates the independence assumption underlying meta-analysis and inflates both the effective sample size and the weight given to this study.

The *PLOS One* Editors sought advice from an independent expert and a Statistical Advisor with expertise in systematic reviews and meta-analyses regarding the concerns outlined above. Both agreed with the Editors’ conclusion that the validity and reliability of the published results are in question. In light of this assessment, the *PLOS One* Editors retract this article.

XL, SG, and GH responded but expressed neither agreement nor disagreement with the editorial decision. JW either did not respond directly or could not be reached.
